# Serum meprin α levels for the detection of systemic inflammatory response syndrome

**DOI:** 10.1186/s10020-026-01570-w

**Published:** 2026-07-18

**Authors:** Silje Beckinger, Marion Mengel, Matthias Lindner, Vasco Köhling, Florian Peters, Inez Götting, Johanna Stoske, Jannik Rusch, David I. Radke, Dirk Schädler, Cynthia Bülck, Kira Bickenbach, Malina Rüffer, Reiner K. Mailer, Neele Schumacher, Thomas Renné, Michael Haase, Ronald Naumann, Christoph Becker-Pauly, Sascha Rüffer

**Affiliations:** 1 Biochemical Institute, Christian-Albrechts-University of Kiel, Kiel, Germany; 2 Institute of Clinical Chemistry and Laboratory Medicine, University Medical Center Hamburg-Eppendorf, Hamburg, Germany; 3 Department of Anaesthesiology and Intensive Care Medicine, University Medical Center Schleswig-Holstein, Kiel, Germany; 4 InnoMedica, Bern, Switzerland; 5 Institute for Pharmacology and Toxicology, Freie Universität Berlin, Berlin, Germany; 6 School of Pharmacy and Biomolecular Sciences, Irish Centre for Vascular Biology, Royal College of Surgeons in Ireland, Dublin, Ireland; 7 Department of Pediatric Surgery, Faculty of Medicine and University Hospital Carl Gustav Carus, TU Dresden, Dresden, Germany; 8 Transgenic Core Facility, Max Planck Institute for Molecular Cell Biology and Genetics, Dresden, Germany

**Keywords:** Systemic inflammatory response syndrome, Meprin α, Diagnostic marker, Intensive care patients

## Abstract

**Background:**

Systemic inflammatory response syndrome (SIRS) is a frequent critical condition in clinical patients marked by dysregulated immune activation and high mortality. Early initiation of appropriate interventions are important for patient outcome, but molecular markers for diagnosis are not SIRS-specific.

**Methods:**

We performed hematological analyses and health-status assessments on a transgenic disease mouse model that recapitulates elevated epidermal levels of the metalloprotease meprin α (K5Mα) reported in inflammatory skin diseases. In a cohort of intensive care patients that either developed SIRS (*n* = 19) or not (*n* = 29), we measured parameters associated with systemic inflammation and organ function as well as serum meprin α levels.

**Results:**

K5Mα mice developed fatal SIRS characterized by hypothermia, severe weight loss, hypochromic microcytic anemia, neutrophilic leukocytosis and cytokine release syndrome. Serum concentrations of meprin α correlated with disease progression in K5Mα mice. We detected high meprin α levels in the serum of intensive care patients who developed SIRS but in none of the patients who did not develop SIRS. Serum meprin α levels significantly correlated with clinical parameters like C-reactive protein, procalcitonin and white blood cell count, but unlike all other measured inflammatory parameters allowed a clear identification of SIRS patients.

**Conclusions:**

We propose serum meprin α levels as a potential biomarker for SIRS. However, we would like to emphasize that due to our limited cohort size subsequent larger-scale, multicentered studies are warranted to validate our findings and potentially provide more detailed insight into whether there is an association between elevated meprin α serum levels and specific causes of SIRS or dysfunction of particular organ systems.

**Graphical Abstract:**

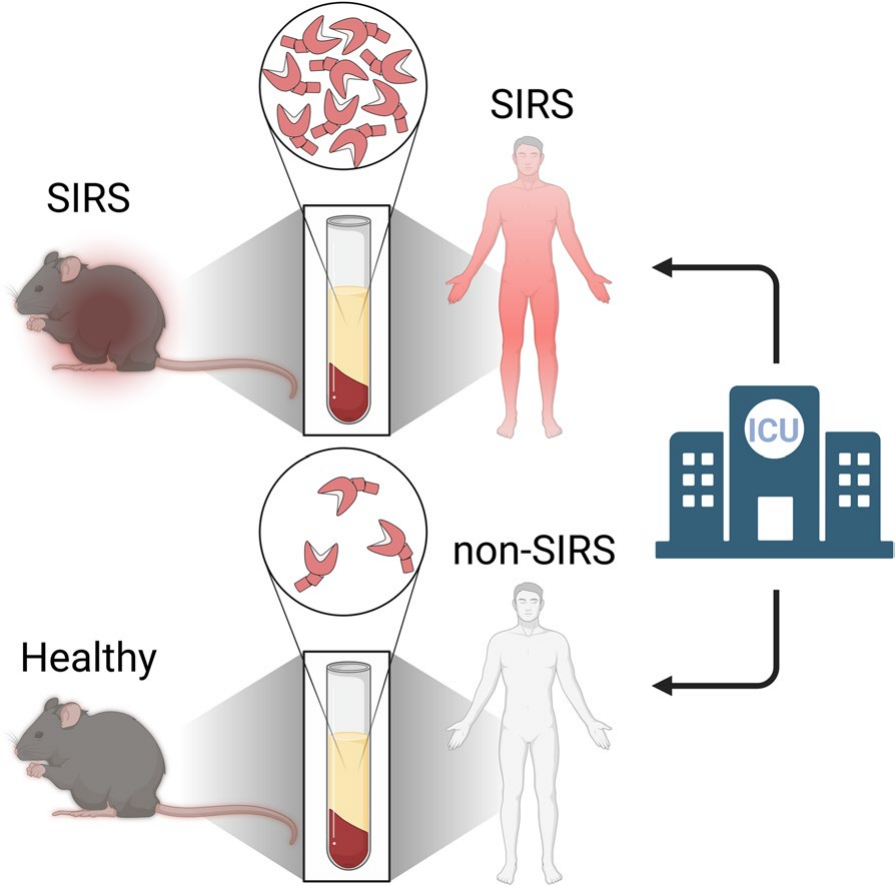

**Supplementary Information:**

The online version contains supplementary material available at 10.1186/s10020-026-01570-w.

## Background

Systemic inflammatory response syndrome (SIRS) is characterized by a detrimental excessive immune response involving multiple organ systems. SIRS can develop from various causes, e.g. infections (bacterial, viral, mycotic and parasitic), acute and chronic inflammatory diseases as well as trauma, surgery and cancer, but also certain medications like immunotherapy and various forms of intoxication (Chakraborty and Burns 2023). Often SIRS develops from distinct foci like pneumonia, cystitis, pancreatitis, infected wounds or locally restricted trauma, from where the inflammatory response spreads to other organ systems and exaggerates (Chakraborty and Burns 2023; Papadopoulou et al. 2014; Dharap and Ekhande 2017). The onset of SIRS is associated with a hyperactive state of the immune system characterized by the activation of leukocytes and the acute phase system accompanied by cytokine release syndrome (Matsuda and Hattori 2006). SIRS is a very frequent complication in hospitals especially faced by intensive care units and depending on its severity can lead to multiple organ dysfunctions (Matsuda and Hattori 2006). Particularly infection-based forms of SIRS, sepsis and septic shock, are associated with a mortality of 21.5 to 41.5% even in Western countries and, therefore, represent major health care issues (Matsuda and Hattori 2006). Early diagnosis of SIRS is critical for patient outcome. However, diagnosis is difficult because inflammatory mediators and symptoms associated with disease onset and progression are not specific. As timely diagnosis is essential for initiation of appropriate treatment (Cavaillon and Adrie 2009; Uffen et al. 2021), biomarkers that specifically indicate the disease onset and progression would be of utmost importance for the outcome of SIRS patients.

Meprin α is a metalloprotease that belongs to the astacin family with a characteristic preference for acidic amino acids around the scissile bond (Becker-Pauly et al. 2011). It is highly expressed in the brush border of the intestine and the kidney as well as the basal layer of the epidermis (Bankus and Bond 1996; Bond et al. 2005; Becker-Pauly et al. 2007) as a multi-domain zymogen activated by tryptic proteases like trypsin, plasmin and kallikrein-related peptidase 5 (Becker et al. 2003; Rösmann et al. 2002; Ohler et al. 2010). In contrast to meprin β, meprin α loses its transmembrane domain on the secretory pathway due to furin cleavage. Therefore, it is typically secreted as homodimers that can further assemble into multimers of several megadaltons in size, or it can be tethered to the plasma membrane in a heterodimeric complex with meprin β (Marchand et al. 1995; Peters et al. 2019; Bayly-Jones et al. 2022). The substrate spectrum of meprin α includes numerous proteins that are essential for the regulation of inflammatory processes. These include cytokines such as IL-1β (Herzog et al. 2009), cytokine receptors such as the IL-6 receptor (Arnold et al. 2017), as well as chemokines, adhesion proteins, and components of the extracellular matrix (Broder and Becker-Pauly 2013). Indicating a mechanistic link to inflammatory diseases, aberrant meprin α levels have been reported in Kawasaki disease, inflammatory bowel disease and psoriasis vulgaris (Becker-Pauly et al. 2007; Banerjee et al. 2009; Kentsis et al. 2013). In addition, mouse models provide evidence that meprin α activity not only contributes to kidney damage during inflammatory processes (Herzog et al. 2007), but may, under certain conditions, also represent a therapeutic target for protecting the renal microcirculation (Wang et al. 2011). We recently showed in a transgenic disease mouse model (K5Mα) that locally elevated epidermal meprin α expression causes a severe inflammatory ichthyosis initiated by degradation of dermokine (Köhling et al. 2026). Unexpectedly, we found that systemically-induced K5Mα mice develop a lethal phenotype and hypothesized that mortality is caused by progression of the inflammatory skin disease into a systemic inflammatory disorder. In the present study, we aimed to characterize development of the lethal phenotype in K5Mα mice with regard to pathological mechanisms and parameters relevant for the assessment of SIRS. Since serum and urine levels of meprin α have been reported to be elevated in Kawasaki disease, a systemic vasculitis of unknown etiology, we additionally investigated if meprin α levels in our mouse model and intensive care patients correlate with markers associated with systemic inflammatory responses.

## Results and discussion

### K5Mα mice develop a lethal SIRS

**Fig. 1:**
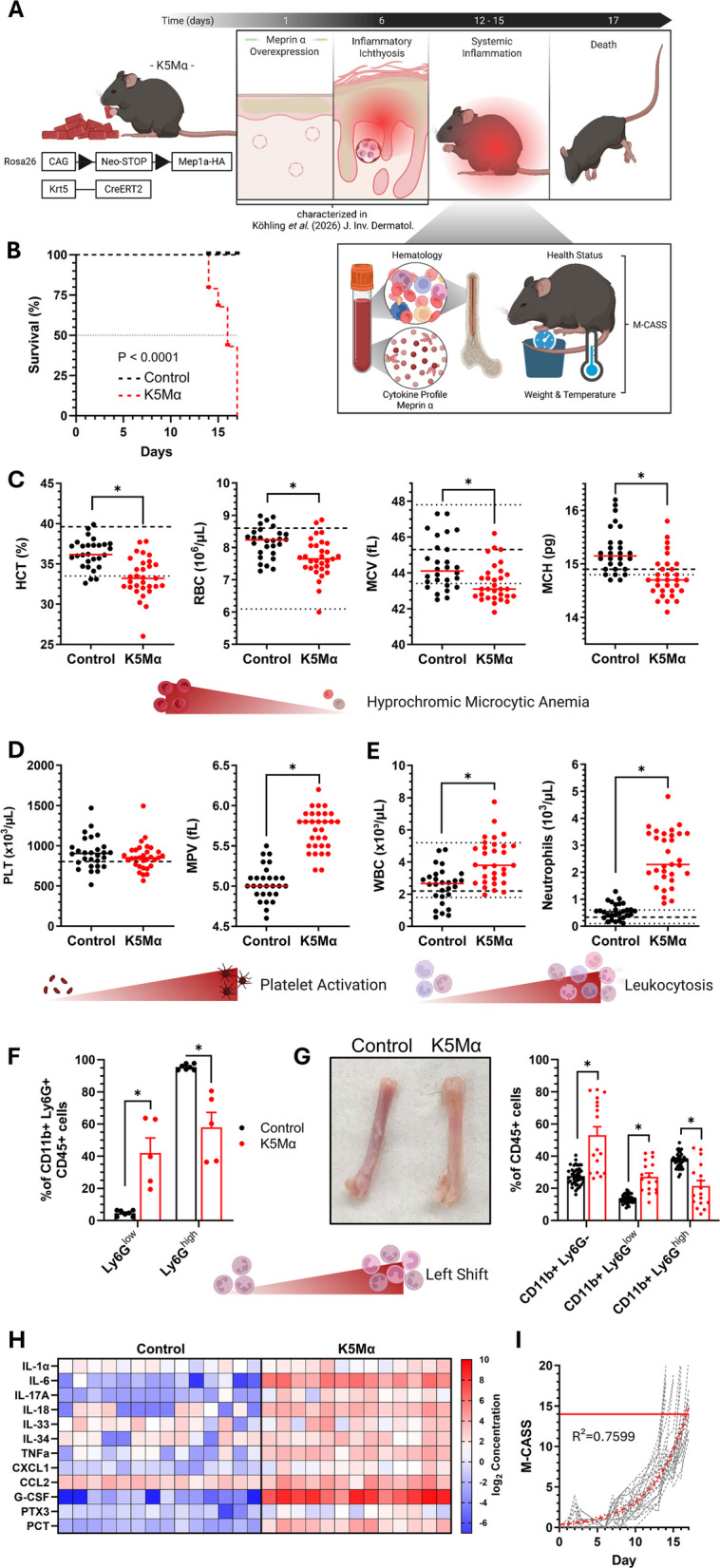
K5Mα mice develop a lethal SIRS. **A** Phenotype development in K5Mα mice after feeding tamoxifen-supplemented food. Skin phenotype has been characterized previously (Köhling et al. 2026). **B** Kaplan–Meier survival analysis of control and K5Mα mice (*n* = 19 *vs*. 19). **C** Hematokrit (HCT), red blood cell count (RBC), mean corpuscular volume (MCV) and mean corpuscular hemoglobin levels indicate development of hypochromic microcytic anemia in K5Mα mice. **D** Platelet count (PLT) and mean platelet volume (MPV) indicate platelet activation in K5Mα mice. **E** White blood cell count (WBC) and neutrophilic leukocyte count reveal neutrophilic leukocytosis in K5Mα mice. **C-E** Data points show respective values detected in individual control (*n* = 28) and K5Mα (*n* = 31) mice. Median values are marked by horizontal red drawn-through lines. Reference ranges are marked by dotted lines and reference mean values by dashed lines. **F** Proportions of premature (Ly6G^low^) and mature (Ly6G^high^) neutrophils in the blood of K5Mα (*n* = 23) and control mice (*n* = 13) show a left shift in K5Mα mice. **G** Femur from a control and K5Mα mouse. Proportions of premature and mature myeloid leukocytes in the bone marrow of K5Mα (*n* = 17) and control (*n* = 42) mice. **F + G** Bar charts represent mean ± SEM. **H** Concentrations of inflammation-associated mediators in the serum of control (*n* = 14) and K5Mα (*n* = 13) mice. IL-1α (pg/ml), IL-6 (pg/ml), IL-17A (pg/ml), IL-18 (× 10^1^ pg/ml), IL-33 (pg/ml), IL-34 (× 10^1^ pg/ml), TNFα (pg/ml), CXCL1 (× 10^2^ pg/ml), CCL2 (pg/ml), G-CSF (× 10^2^ pg/ml), PTX3 (× 10^2^ ng/ml), PCT (ng/ml). **I** Progression of the Modified Mouse Clinical Assessment Score for Sepsis (M-CASS) in K5Mα mice (*n* = 26). Non-linear regression (red dashed line) and respective 95% confidence band of the best-fit line (red dotted lines). M-CASS threshold for euthanasia by predetermined humane endpoint criteria (–-). Statistically significant differences (*P* < 0.05) are labeled by an asterisk (*). Graphical illustrations were created with BioRender.com. M-CASS, Modified mouse clinical assessment score for sepsis

K5Mα mice were fed a tamoxifen-supplemented diet for induction of meprin α overexpression at the age of 8 to 20 weeks. Besides the inflammatory ichthyosis previously described (Köhling et al. 2026), we observed that all K5Mα but none of the control mice showed a rapidly worsening of their overall condition (Fig. 1A). Hence, within 17 days after start of the tamoxifen diet, all K5Mα mice had to be taken out of the experiment by euthanasia and some even spontaneously died (Fig. 1B). Since we suspected that K5Mα mice died due to a severe systemic inflammatory response, we examined the mice for inflammation-associated changes (Fig. 1A). Indeed, K5Mα mice developed hypochromic microcytic anemia (Fig. 1C, Fig. S1A), showed signs of platelet activation (Fig. 1D) and a prominent leukocytosis (Fig. 1E) characterized by lymphopenia (Fig. S1B + C), neutrophilia (Fig. 1E, Fig. S1C), elevated proportion of inflammatory monocytes (Fig. S1C) and a left shift (Fig. 1F) associated with emergency granulopoiesis (Fig. 1G, Fig. S1D) potentially driven by G-CSF (Fig. S1E). Levels of inflammatory cytokines and chemokines in the blood were drastically elevated (Fig. 1H), including procalcitonin, a marker of systemic inflammation (Castelli et al. 2004). Moreover, serum from K5Mα, but not control mice, strongly activated naive neutrophilic granulocytes (Fig. S1F). Further assessments by behavioral observations and physical examinations based on adapted scoring systems reported by Mai and colleagues for monitoring of septic mice (Mai et al. 2018) as well as Langford and colleagues for monitoring of pain (Langford et al. 2010) (for detailed description see Material and Methods; Fig. S1G) underscored the rapid drastic deterioration of the animals ‘ health condition within only one to three days. Here, a modified Mouse Clinical Assessment Score for Sepsis (M-CASS) of 14 met our predetermined humane endpoint criteria as a predictive value for high risk of mortality (Fig. 1I). In our previous study, where we introduced the mouse model and thoroughly characterized the development of the inflammatory skin phenotype, we identified several inflammatory mediators that may explain how a local skin inflammation might develop into a systemic inflammatory syndrome (Köhling et al. 2026). Combined with the data in the present study, we propose that SIRS in K5Mα mice develops by the following cascade. Dermokine, which is inactivated by meprin α, not only functions as a negative regulator of keratinocyte proliferation (Higashi et al. 2012; Utsunomiya et al. 2020) but also of ELR + CXC chemokine expression (Hasegawa et al. 2013). Hence, meprin α-mediated degradation of dermokine initiates the recruitment of neutrophils and monocytes, which start the inflammatory cascade observed in the skin of K5Mα mice (Köhling et al. 2026). In the skin we found highly elevated levels of GM-CSF, which is released by keratinocytes under stress and inflammatory conditions. GM-CSF acts as potent driver of M1-like macrophage polarization (Mann et al. 2001; Mantovani et al. 2013). Consequently, macrophages derived from recruited monocytes foster the inflammatory reaction, e.g. by the secretion of TNFα, IL-6 and IL-18, that spread the inflammatory response to fibroblasts and endothelial cells (Chen et al. 2023). Macrophages, endothelial cells and fibroblasts are key producers of G-CSF (Chang et al. 2015; Boettcher et al. 2014; Kaushansky et al. 1988), which induces granulopoiesis in the bone marrow to maintain sufficient production of myeloid cells (Roberts 2005). Since K5Mα mice develop the inflammatory ichthyosis across their entire skin, this inflammatory cascade would be a reasonable explanation for the G-CSF levels, which were up to 1,500-fold elevated in the serum of K5Mα compared to control mice. In turn, these drastically elevated G-CSF levels could be causative for the emergency granulopoiesis observed in the bone marrow, which then again causes neutrophilia as well as altered erythropoiesis and hemoglobin synthesis (Roberts 2005; Martin et al. 2021). In order to test this hypothesis, we treated K5Mα mice with a G-CSF neutralizing antibody (Morris et al. 2015; Jing et al. 2021) (Fig. S2A). Although G-CSF levels were only modestly reduced upon antibody treatment (Fig. S2B), blood analyses revealed decreased numbers of leukocytes (Fig. S2C), especially neutrophils (Fig. S2D), as a consequence of G-CSF-neutralization. The strongest effects, however, were observed in the myeloid compartment of the bone marrow (Fig. S2E). Together, these results support the hypothesis that pathologically elevated G-CSF levels contribute causally to the dysregulated granulopoiesis in K5Mα mice. Overall, we showed that K5Mα mice develop SIRS as evidenced by neutrophilic leukocytosis with a left shift (Chakraborty and Burns 2023; Copeland et al. 2005), hypochromic microcytic anemia (Darveau et al. 2004; Valdés-Ferrer et al. 2015), severe hypothermia (Mei et al. 2018; Tan et al. 2024) and cytokine release syndrome with highly elevated PCT levels. These features represent key hallmarks of systemic inflammation (Nie et al. 2025; Eskandari et al. 1992; Beurel and Jope 2009; Gharamti et al. 2022; Vijayan et al. 2017; Zaki et al. 2024) that are central characteristics of both murine SIRS models and SIRS patients. Unfortunately, we were not able to correlate lethality of K5Mα mice with the dysfunction of a specific organ (data not shown). This might be attributed to the fact that we had to adapt our humane endpoint criteria and monitoring protocol in order to minimize phenotype-associated stress and prevent spontaneous death. A possible cause for organ failures could be coagulation disorders due to the inflammatory systemic activation of neutrophils and platelets, which lead to vascular occlusions (Korniluk et al. 2019).

### Meprin α serum levels correlate with lethal phenotype progression

**Fig. 2:**
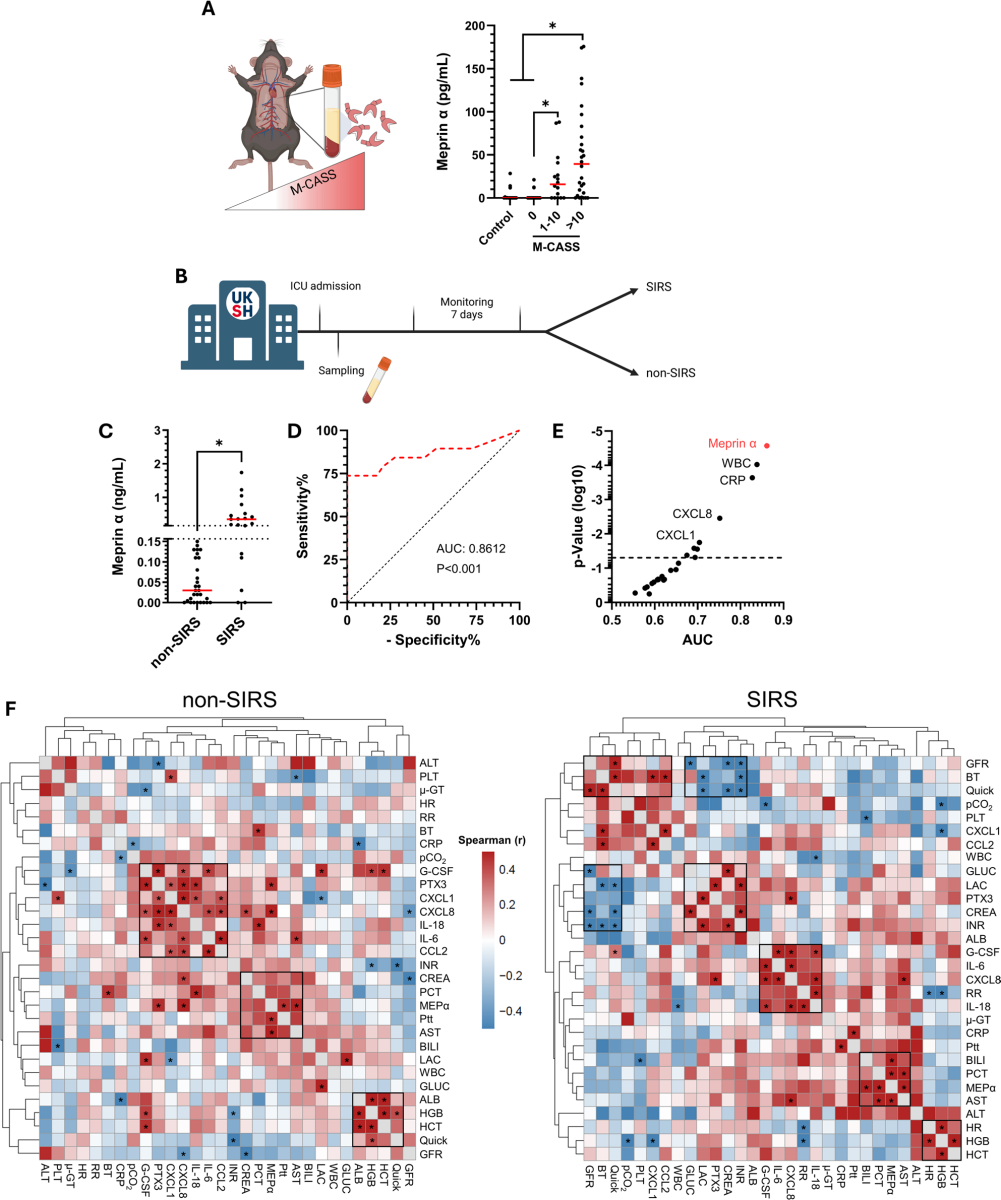
Serum meprin α concentrations correlate with SIRS development in K5Mα mice and discriminate SIRS from non-SIRS patients. **A** Serum meprin α concentrations in control mice and K5Mα mice grouped by M-CASS. Median values are marked by horizontal red drawn-through lines. **B** Schematic illustration of the clinical study design. **C** Serum meprin α levels in non-SIRS and SIRS patients. Median values are marked by horizontal red drawn-through lines. Dotted line marks the lowest standard concentration used in the ELISA. **D** Receiver Operating Characteristic analysis by Wilson/Brown method indicating sensitivity and specificity of serum meprin α concentrations for discrimination of SIRS from non-SIRS patients. **E** Results of Receiver Operating Characteristic analyses by Wilson/Brown method for discrimination of non-SIRS from SIRS patients by clinical parameters. **F** Spearman correlation matrix for clinical parameters in non-SIRS and SIRS patients sorted by Euclidean distance clustering. Statistically significant correlations are marked by an asterisk (*) and clusters are illustrated by dendrograms and boxes. Graphical illustrations in (**A + B**) were created with BioRender.com. ALB, albumin; ALT, alanine amino transferase activity; AST, aspartate amino transferase activity; AUC, area under the curve; BILI, bilirubin; BT, body temperature; CCL2, chemokine ligand 2; CREA, creatinine; CRP, C-reactive protein; CXCL1/8, C-X-C motif chemokine ligand 1/8; µ-GT, gamma glutamyl transferase activity; G-CSF, granulocyte colony-stimulating factor; GFR, glomerular filtration rate; GLUC, glucose; HCT, hematocrit; HGB, hemoglobin; HR, heart rate; ICU, intensive care unit; IL, interleukin; INR, international normalized ratio; LAC, lactate; M-CASS, modified mouse clinical assessment score for sepsis; MEPα, meprin α; pCO_2_, partial pressure of carbon dioxide; PCT, procalcitonin; PLT, platelet count; Ptt, partial thromboplastin time; PTX3, pentraxin-3; RR, respiratory rate; SIRS, systemic inflammatory response syndrome; WBC, white blood cell count

Since Kentsis and colleagues reported elevated meprin α levels in the serum and urine of Kawasaki disease patients and a corresponding mouse model (Kentsis et al. 2013), and Kawasaki disease shares many pathophysiological aspects of SIRS (Tomobe et al. 2019), we asked whether meprin α is also detectable in K5Mα mice. Strikingly, while serum meprin α concentrations in control and K5Mα mice with a M-CASS of 0 were either very low or even undetectable, we found significantly elevated meprin α levels in K5Mα mice that had developed an advanced phenotype (Fig. 2A). In order to test whether elevated serum meprin α levels were a consequence or even mechanistically involved in the phenotype progression observed in K5Mα mice, we generated mice that constitutively overexpress meprin α under control of the *Lyz2* promoter (*Lyz2*^*tm1(Cre)lfo*^;*Rosa26*^Stop−*mMep1a*^, abbreviation: LMα), which is highly active in neutrophils and monocytes (Fig. S3A). As expected, high meprin α levels were detectable in the bone marrow (Fig. S3B), but we did not observe any pathological changes indicative for an inflammatory response (Fig. S3C-U). Ultimately, it cannot be determined where the elevated serum meprin α levels in K5Mα mice originate from. One possibility is that meprin α enters the bloodstream through the skin, whose barrier function is compromised by the inflammatory phenotype. Evidence against this hypothesis comes from the observation that not all mice with a terminal phenotype display such high meprin α serum levels, while elevated concentrations are already detectable in mice that have not developed severe skin lesions. Alternatively, meprin α may originate from other organs with high protein expression, whose functionality and barrier integrity are affected by systemic inflammatory syndromes—a topic that we will discuss further in the following section.

### Meprin α serum levels enable discrimination of SIRS from non-SIRS intensive care patients and correlate with clinical parameters

In order to investigate whether serum meprin α levels also correlate with development of SIRS in humans, we monitored patients who had been admitted to the intensive care unit (ICU) of the University Medical Center Schleswig–Holstein with suspected infection and an indication for antibiotics for seven days in a prospective study. We measured clinical parameters and serum meprin α levels after admission to the ICU and monitored patients for seven days to identify patients who developed SIRS (Fig. 2B). In total, 19 of 48 patients developed a SIRS during their stay at the ICU (Table 1).

**Table 1:** Study cohort characteristics. Patients admitted to the intensive care unit (ICU) at the University Medical Center Schleswig–Holstein were monitored for seven days. Patients included in the study had a suspected infection with an indication for antibiotic treatment. Grouping was based on development of a systemic inflammatory response syndrome (SIRS) during stay at the ICU. Mortality was monitored within 28 days after study inclusion

Parameter	Non-SIRS	SIRS
Number	29	19
Median Age (min/max)	66 (45/87)	66 (29/84)
Female (%)/Male (%)	10 (34.5)/19 (65.5)	6 (31.5)/13 (68.5)
Surgical (%)	16 (55.1)	13 (68.5)
Deceased (%)	6 (20.7)	11 (57.9)

While meprin α levels in sera of all patients who did not develop SIRS were under the detection limit, meprin α was detectable (concentration higher than that of the lowest standard) in the serum of 14 out of 19 SIRS patients with a median concentration of 0.35 ng/mL and up to 1.74 ng/mL (Fig. 2C; Table S1). Interestingly, we found significant correlations between serum meprin α levels and many inflammation-associated parameters, e.g. CRP, PCT, PTX3, WBC, IL-6, IL-8, IL-18, as well as markers associated with organ dysfunction, e.g. AST, GFR, creatinine, bilirubin and µ-GT (Table S1). Overall, serum meprin α levels were superior for discrimination of non-SIRS from SIRS patients compared to any other clinical parameter examined in our study (Fig. 2D + E; Table S1). Notably, six of the 19 SIRS patients did not meet the SIRS criteria at the time of admission to the ICU, but already exhibited elevated meprin α levels, suggesting that serum meprin α levels may already indicate the risk of developing SIRS.

Finally, we asked whether there are substantial differences in the correlations of clinical parameters between our two patient groups (Fig. 2F). Hierarchical clustering of the correlation matrix showed that within the non-SIRS group cytokines and chemokines formed a large isolated cluster, whereas in the SIRS patient group they were found in clusters with vital parameters or markers of organ dysfunction. Interestingly, serum meprin α levels were located in a cluster with PCT and AST in both the non-SIRS and the SIRS group. All in all, these data suggest that serum meprin α levels not only reflect the development of SIRS but may also indicate the progression of organ dysfunction in this context. Meprin α is highly expressed in the proximal tubules of kidney and the brush border of the intestine (Bond et al. 2005). Since inflammation-mediated renal injury and dysfunctional intestinal barrier are commonly observed in SIRS (Chancharoenthana et al. 2023; Tsalik et al. 2015; Vitorio 2014), serum meprin α might originate from the kidneys or the intestine. In K5Mα mice we did not find evidence of impaired kidney function (serum creatinine levels, data not shown) but in our patient cohort we identified a significant positive correlation between serum meprin α levels and glomerular filtration rate as well as levels of creatinine, bilirubin and µ-GT. To determine whether serum meprin α levels might be attributable to renal leakage, we measured protein concentrations in patients’ urine samples. Interestingly, meprin α was detectable in seven out of ten urine samples; however, differences in concentrations were only apparent as a trend (Fig. S4A). Since meprin α and its genetically close relative meprin β are synthesized at high levels at the brush border in the proximal tubular system of the kidney (Bond et al. 2005), the presence of meprin α in the urine is not necessarily associated with pathology (Herzog et al. 2007; Beynon et al. 1981). However, elevated meprin α levels in the urine have been reported in patients with Kawasaki disease (Kentsis et al. 2013), a finding that might be attributed to either an inflammation-induced upregulation of its expression and/or increased shedding of the heterodimeric complex formed with meprin β (Peters et al. 2019) by proteases like ADAM10 or ADAM17, which are also regulated by inflammatory processes (Kato et al. 2018; Herzog et al. 2014). Interestingly, the highest meprin α concentration was detected in the urine of a patient from the non-SIRS group (Fig. S4A). Of note, this very patient was the only individual who had been admitted to the intensive care unit following cardiac surgery involving the use of cardiopulmonary bypass, a procedure that causes a strong systemic inflammatory response. Whether this specific surgical procedure leads to increased meprin α concentrations in serum and urine could be investigated in subsequent studies.

Alternatively, meprin α might be secreted by other cell populations involved in or affected by the inflammatory cascade, e.g. endothelial cells or leukocytes (Bond et al. 2005). However, while in endothelial cells there is currently only indirect evidence (Biasin et al. 2018), as well as unpublished observations, suggesting the expression of meprin α, the evidence for its expression in leukocytes is based solely on analyses of mesenteric lymph nodes from mice (Crisman et al. 2004). Our own attempt to detect meprin α expression in leukocytes from non-SIRS and SIRS patients showed that meprin α mRNA levels were detectable in only two out of ten patients, and even then, only at very low levels (Ct-values > 30) (Fig. S4B). Overall, these findings suggest that MEP1A is neither constitutively expressed by human leukocytes nor that its expression in leukocytes is regulated by systemic inflammatory processes. Since also highly elevated meprin α levels in mice that overexpressed meprin α in myeloid cells were not associated with development of systemic inflammation, we assume that elevated serum levels of meprin α are not a driver of SIRS but rather a consequence. Over the past years, we and others have generated numerous mouse models in which the expression levels of meprins were modulated in order to identify both physiologically and pathologically relevant substrates. Thereby, we found that the presence of endogenous activators and inhibitors in the tissue has a major impact, and that elevated levels alone are not necessarily functionally relevant. The absence of a phenotype in the LMα mice is likely explained by the fact that proteolytic activities in the blood are tightly regulated by various inhibitors, such as Fetuin-B, a very potent meprin inhibitor (Karmilin et al. 2019). As a potential function of serum meprin α under inflammatory conditions, Biasin and colleagues provided evidence that binding of meprin α to heparan sulfates of the endothelial glycocalyx limits neutrophil extravasation in idiopathic pulmonary arterial hypertension patients, indicating that meprin α limits the spread of inflammation by a non-proteolytical mechanism (Biasin et al. 2018). However, if meprin α exhibits proteolytic activity in endothelial cells, then the shedding of the IL-6 receptor—which promotes pro-inflammatory IL-6 trans-signaling—would represent a mechanism, which has been proposed as a therapeutic target in SIRS and many other inflammatory diseases (Barkhausen et al. 2011; Rose-John et al. 2023).

## Conclusions

Overall, we think that our findings are significant for several reasons. Various mouse models, which aimed to modulate the expression or activity of meprin α and meprin β in the intestine (Wichert et al. 2017; Bülck et al. 2023), the kidney (Bond et al. 2005; Kaushal et al. 2013), the brain (Marengo et al. 2022; Armbrust et al. 2019, 2024) and the skin (Kruppa et al. 2021; Peters et al. 2021) have been generated in the last years to investigate whether altered meprin levels are causally linked to inflammatory and neurodegenerative diseases (Bond et al. 2005; Banerjee et al. 2009). Here, we report a clear functional role of meprin α in systemic inflammatory processes, which has been suggested by many studies but never validated in vivo before. Moreover, we showed that meprin α can initiate inflammatory inter-organ crosstalk, which is particularly relevant for understanding the complexity of systemic inflammatory syndromes, and provided evidence for an underlying signaling cascade. Lastly, and from a translational perspective most relevant, we found a correlation between SIRS development and elevated meprin α levels in the serum of both K5Mα mice and intensive care patients. Importantly, our study emphasizes that serum meprin α could be a clinically useful marker that is highly specific for the diagnosis of SIRS. Nonetheless, we would like to highlight as a limitation of our study that our patient cohort consisted of only 48 patients. Therefore, further studies with larger cohorts should evaluate whether meprin α levels in sera from SIRS or even sepsis patients might be utilized as a biomarker for diagnosis, monitoring and/or prognosis to improve patient treatment and outcome.

## Material and methods

### Study design

In this study a transgenic mouse model and human intensive care patients were investigated. After induction of epidermal meprin α overexpression, the mouse model C57BL/6 J-KRT5^tm1.1−CreERT2^;ROSA26^Stop−mMep1a^ (K5Mα) developed a lethal phenotype on the basis of a hyperproliferative inflammatory skin disease that has been described previously (Köhling et al. 2026). We hypothesized that lethality was caused by a systemic inflammatory response syndrome. Therefore, we characterized phenotype development in K5Mα mice with a focus on physiological parameters relevant in SIRS. An ANOVA with fixed effects (dF = 3, α error probability = 0.05, Cohen effect size = 0.8) was performed using the G*Power program to calculate the required number of animals per group and for statistical analysis (Faul et al. 2007). Animals with obvious malformations (e.g., enlarged testicles, blindness) and injuries or diseases that could be associated with inflammatory processes were excluded from the experiment. Data from animals in whom such malformations (e.g., urinary tract obstruction) or injuries and diseases that were not phenotype-specific were observed during the experiment or after euthanasia were not included in the analysis. As endpoints of the experimental observation period, stopping criteria were defined in the underlying animal experiment protocol, which was approved by the ministry for Agriculture, Rural Areas, Europe and Consumer Protection of the state Schleswig–Holstein (reference V242—12,659/2018 (30–4/18)) before the study was initiated.

Patients were recruited from November 2023 to May 2024 at the University Medical Center Schleswig–Holstein in Germany for the prospective study. Included were full-aged patients (> 18 years) admitted to the intensive care unit with suspected infection and indication for antibiotic treatment after informed consent has been given. All patient data were pseudonymized prior to study inclusion. Serum samples were taken after admission to the ICU as well as on day one, four and seven for quantification of meprin α concentration. Additionally, whole blood samples for leukocyte isolation and urine samples were collected from five non-SIRS and five SIRS patients after confirmed diagnosis. Patient sera were analyzed for meprin α levels with the hypothesis that meprin α levels are elevated in the serum of SIRS compared to non-SIRS patients. Routine laboratory diagnostics were performed by Institute of Clinical Chemistry at the University Medical Center Schleswig–Holstein. Excluded were pregnant and breastfeeding women as well as patients who participated in an interventional study within the last 30 days. No study-specific or therapeutic interventions were conducted. All patients received the best possible standard therapy for their underlying condition. At hospital discharge before day 28, the survival status of the participating patients was recorded. If discharge occurred before day 28, survival status was obtained for patients still in the hospital via the hospital internal information system, and for participants already discharged via a telephone visit. Documentation from the electronic patient record included demographic variables and patient characteristics, as well as treatment course and vital parameters on the day of operation and during the stay in intensive care. Approval was granted by the Ethics Committee of the Medical Faculty of the Christian-Albrechts University (reference number: D546/23). Study details are accessible via the German Clinical Trial Register (reference number: DRKS00032213).

### Chemicals

All chemicals were purchased of analytical grade from Merck, Carl Roth, Thermo Fisher Scientific or Roche.

### Animal care

Animal acquisition, breeding, accommodation, care and usage in experimental procedures was performed in accordance with the guidelines of the German National Committee for the Protection of Animals Used for Scientific Purposes as previously reported (Köhling et al. 2026). Animals were housed under specific pathogen-free conditions in an individually ventilated cage system at constant environmental conditions (12 h light–dark cycle, 22 ± 2 °C, 55 ± 10% relative humidity). A maximum of five male or female siblings were housed in one cage with food and drinking water supply ad libitum. Cages were enriched with aspen litter, houses and tubes. Daily animal care was performed by professional animal care attendants.

### Generation of C57BL/6 J-KRT5^tm1.1−CreERT2^;ROSA26^Stop−mMep1a^ and C57BL/6 J-Lyz2^tm1(Cre)lfo^;Rosa26^Stop−mMep1a^ mice

Meprin α knock-in mice (C57BL/6 J-ROSA26^Stop−mMep1a^) were generated at the Transgenic Core Facility of the Max Planck Institute for Molecular Cell Biology and Genetics in Dresden, Germany, as reported (Köhling et al. 2026). Murine meprin α cDNA (Transcript ID: ENSMUST00000117137.8; A95-G2335) was cloned into pCAG-ROSA-IRES-EGFP targeting vector. Murine JM8A1.N3 embryonic stem cells (Pettitt et al. 2009) were transfected by electroporation and clones selected that incorporated the transgenic construct (CAG-loxP-Neo^R^-STOP-loxP-mMep1a-HA-IRES-GFP) by homologous recombination into the Rosa26 locus of their genome. Suitable embryonic stem cell clones were transplanted by laser-assisted microinjection into C57BL6/NCrl morula stages 2.5 days post coitum (Poueymirou et al. 2007) for fertilization of recipient female mice. Sperms of chimeric male offspring were screened by short tandem repeat analysis for transgenic proportion and suitable samples were selected for in vitro fertilization. Heterozygous offspring was crossed with B6N.129S6(Cg)-Krt5^tm1.1(cre/ERT2)Blh^/J mice (Keymeulen et al. 2011) (Jackson Laboratory, stock #029155) to generate C57BL/6 J-KRT5^tm1.1−CreERT2^;ROSA26^Stop−mMep1a^ mice (patent application EP20160475.8/W0 2021/175738 A1). Mice that carried homozygous the mMep1a construct within the Rosa26 locus and either i) heterozygous the information for the Cre recombinase/modified estrogen receptor fusion protein (CreER^T2^+/-; K5Mα) or ii) not (CreER^T2^-/-; Control) were used for subsequent experiments. In order to breed only mice with a heterozygous and not homozygous CreER^T2^ status, CreER^T2^-positive mice were only crossed with CreER^T2^-negative mice.

For generation of Lyz2^tm1(Cre)lfo^;Rosa26^Stop−mMep1a^ mice, C57BL/6 J-ROSA26^Stop−mMep1a^ were crossed with B6.129P2-Lyz2^tm1(cre)lfo^/J mice (Clausen et al. 1999) (Jackson Laboratory, stock #004781). Mice that carried homozygous the mMep1a construct within the Rosa26 locus and either i) heterozygous the information for the Cre recombinase (Cre +/-; LMα) or ii) not (Cre-/-; Control) were used for subsequent experiments.

### Genotyping

Genotyping of C57BL/6 J-KRT5^tm1.1−CreERT2^;ROSA26^Stop−mMep1a^ and C57BL/6 J-Lyz2^tm1(Cre)lfo^;Rosa26^Stop−mMep1a^ mice was performed as reported (Köhling et al. 2026). Genomic DNA was extracted from ear punches using the DirectPCR® Lysis Reagent Tail (Peqlab) according to the manufacturers’ instructions. For subsequent PCR reaction 2 µL lysate was added to a total reaction volume of 30 µL containing DreamTaq DNA polymerase (80 IU/mL), 3 µL DreamTaq Green Buffer (10X), 2 mM dNTPs and respective primers at a concentration of 0.4 µM. Analysis of the Rosa26 locus zygosity for wildtype (wt) sequence and transgenic (tg) sequence was performed using the following primer set: forward (wt/tg) 5’-aaagtcgctctgagttgttatc-3’; reverse (wt) 5’- gatatgaagtactgggctctt-3’; reverse (tg) 5’- tgtcgcaaattaactgtgaatc-3’. PCR products at a size of 570 bp and 380 bp indicated wt and tg Rosa26 locus, respectively. Analysis of the CreER^T2^ and Cre status was performed using the following primer set: forward 5’- ggttcgcaagaacctgatggacat-3’; reverse 5’- gctagagcctgttttgcacgttca-3’. A PCR product at a size of 342 bp indicated insertion of the CreER^T2^/Cre construct downstream of the keratin 5 and Lyz2 promoter, respectively. The following PCR programs were run for genotyping of Rosa26 and CreER^T2^/Cre status: i) Rosa26: 95 °C for 3 min, 35 cycles (95 °C for 30 s, 56 °C for 30 s, 72 °C for 50 s), 72 °C for 10 min; ii) CreER^T2^: 95 °C for 5 min, 30 cycles (95 °C for 30 s, 56 °C for 30 s, 72 °C for 15 s), 72 °C for 10 min.

### Tamoxifen treatment

Mice were fed tamoxifen-supplemented food for induction of meprin α overexpression. Food pellets supplemented with 400 mg/kg tamoxifen citrate (product ID: TD.130860) were purchased from Inotiv and fed weekly ad libitum from Monday to Friday. Saturday and Sunday mice were fed ad libitum with standard mouse keeping food pellets (Ssniff Spezialdiäten GmbH, Germany).

### G-CSF neutralization

For systemic neutralization of G-CSF, 25 µg of a G-CSF neutralizing antibody (R&D Systems, #MAB414, clone #67604) diluted in sterile saline solution were injected once intraperitoneal. In the control group, the same amount of an isotype control antibody (rat IgG_1_ anti-HRP; InVivoMAb) was injected.

### Modified mouse clinical assessment score for sepsis

Development of the systemic inflammatory response syndrome observed in K5Mα mice upon induction of meprin α overexpression was assessed by a scoring system modified according to Mai and colleagues (Mai et al. 2018). Modified Mouse Clinical Assessment Score for Sepsis (M-CASS) criteria are listed in Table 2.

**Table 2:** Criteria for grading of the modified mouse clinical assessment score for Sepsis. Modified according to Mai et al. (2018)

Parameter/Score	0	1	2	3
Fur condition	Normal coat	Slightly ruffled	Ruffled	Ruffled & grease or piloerection
Activity	Normal	Reduced	Only after provocation	Littler or none after provocation
Posture	Normal	Hunched	Hunched & strained or stiff	Hunched & little/no movement or seizures
Chest movement	Normal	Mild dyspnea	Moderate dyspnea	Severe dyspnea
Eyes	Normal, open	Occasionally closed	Mostly partially closed	Mostly completely closed
Pain (Grimace Scale*)	No	Mild	Moderate	Severe
Body weight	96–100%	90–95%	80–89%	< 80%
Body temperature	36.5–37 °C	36.0–36.4 °C	35.0–35.9 °C	< 35.0 °C

^*^Grimace Scale criteria are listed in Table 3

Relative body weight-loss was calculated based on the body weight measured on day zero of the experiment. Body temperature was measured by infrared thermography using a ThermoScan 7 ear thermometer (Braun, Germany). Grimace scale assessment was modified according to Langford and colleagues (Langford et al. 2010). Criteria are listed in Table 3.

**Table 3:** Criteria for assessment of the pain by Grimace Scale according to Langford et al. (Langford et al. 2010)

Parameter/Score	0	1	2	3
Nose and cheek bulge	Normal, flat	Slightly rounded extension of skin around nose bridge	Wrinkled nose or cheeks, slight bulge in cheeks	Obvious, rigid appearing nose and cheek bulge
Ear positioning	Ears flat, back against the body	Ears alert, slightly angled from back	Ears partially positioned forward or apart	Ears completely erect, far apart
Whisker change	Normal	Some whiskers erect	Whiskers mostly erect or clumping	All whiskers standing on end

Mice that developed a cumulative M-CASS Score of 14 showed a very high probability to die within the following 24 h and, therefore, were immediately taken out of the experiment by euthanasia after reaching our predetermined humane endpoint criteria.

### Anesthesia, euthanasia and whole blood/serum collection

Mice were anesthetized by intra-peritoneal injection of ketamine (120 mg/kg BW) and xylazine (16 mg/kg BW) in sterile PBS. After entering surgical level of tolerance, mice were subjected to thoracotomy and exsanguinated. For anticoagulation whole blood was transferred into EDTA-coated tubes (Sarstedt, Germany). For isolation of serum, whole blood was transferred into anticoagulant-free tubes, incubated at room temperature for 15 min and centrifuged for 10 min at 1,000 g. Serum was stored at −80 °C until analysis.

### Blood count measurement

Blood count measurements were performed with the hematology analyzer Element HT5 (Heska via Antech, Germany). Samples were measured in pre-dilution modus. For this purpose, 20 µL EDTA anti-coagulated whole blood was diluted with 480 µL EHT5 Diluent (Heska via Antech, Germany).

### PCT, PTX3 and meprin α quantification

PCT concentrations in serum samples were measured with the PCT BioAssay™ ELISA Kit (Mouse) (Cat. #383995, Unites States Biological via Biomol, Germany). Pentraxin 3 concentrations in serum samples were measured with the Mouse Ptx3 ELISA Kit (Cat. #RAB0857, Sigma-Aldrich/Merck, Germany). Meprin α concentrations in serum samples of mice were measured with the MEP1a ELISA Kit (Mouse) (Cat. #OKEH03481, Aviva Systems Biology via Biozol, Germany). Meprin α concentrations in serum samples of humans were measured with the MEP1A ELISA Kit (Human) (Cat. #OKDD04731, Aviva Systems Biology via Biozol, Germany). All kits were used according to the manufacturer’s instructions. Measurements were performed with an Infinite® 200 PRO M Plex microplate reader (Tecan).

### Quantification of reactive oxygen species and super oxides

Levels of reactive oxygen species (ROS) and superoxides (SO) in neutrophils were measured by flow cytometry using the ROS/Superoxide Detection Assay Kit (Cell-based) (Cat. #ab139476, Abcam, UK) according to the manufacturer’s instructions. Measurement was performed with a FACSymphony A1 flow cytometer (Becton Dickinson, Germany). Compensation and data analysis were performed with FlowJo software (Becton Dickinson).

### Bone marrow isolation

After euthanasia, thighs of mice were dissected. Then, skin and flesh were removed to expose the femur. The distal and proximal ends of the femur were cut off and the bone marrow was flushed out of the bone with 6 mL cold PBS using a syringe with a 26G × 38'' needle. After dispersion of the bone marrow cells, the suspension was filtered through a 30 μm cell strainer and washed twice with 1 mL cold PBS. The cell number was determined using a Neubauer counting chamber.

### Isolation of neutrophils from bone marrow

Neutrophils were isolated from bone marrow by negative selection with the MojoSort™ Mouse Neutrophil Isolation Kit (Cat. #480058, BioLegend/Revvity, US) according to the manufacturer’s instructions. Resulting neutrophil purity was checked by flow cytometry.

### Sodium dodecyl sulfate polyacrylamide gel electrophoresis

SDS-PAGE was performed with the MINI-Protean Tetra Cell system from Bio-Rad as previously described (Köhling et al. 2026). In preparation lysates were adjusted to an equal protein concentration with respective lysis buffer and diluted 5:1 with 5X SDS-PAGE sample buffer (250 mM Tris–HCl, 350 mM SDS, 7.5 mM bromophenol blue, 50% (v/v) glycerol, 100 mM DTT in ddH_2_O, pH 6.8). Samples were denatured by incubation for 10 min at 95 °C. Discontinuous SDS-PAGE was carried out with a polyacrylamide content of 7.5%, 10%, 12%, 14% or 18% in the separating gel dependent on the desired resolution. Electrophoresis was run at constant voltage of 90 V in the collecting gel and 120 V in the separating gel. PageRuler™ Prestained Protein Ladder (Thermo Fisher Scientific) was used as a molecular weight marker.

### Western blotting

Western blotting was performed with the Criterion™ tank blotting system from Bio-Rad as previously described (Köhling et al. 2026). For the transfer onto a polyvinylidene fluoride membrane in tank blot buffer (25 mM Tris–HCl, 200 mM glycine, 20% (v/v) methanol in ddH_2_O, pH 8.3) a constant current of 2.5 mA/cm^2^ was applied for 2 h at 4 °C. Afterwards, membranes were washed for 5 min with TBS-T (25 mM Tris–HCl, 150 mM NaCl, 0.05% (v/v) Tween-20 in ddH_2_O, pH 7.5) and blocked by incubation in 5% (w/v) skim milk powder in TBS-T for 1 h at room temperature. Then, membranes were incubated overnight on a roller mixer at 4 °C with respective primary antibodies diluted in either 5% (w/v) skim milk powder/TBS-T or 5% (w/v) bovine serum albumin/TBS-T. The following day, membranes were washed twice with TBS-T, once with TBS and incubated for 1 h on a roller mixer at room temperature with horse radish peroxidase-conjugated secondary antibodies in 5% (w/v) skim milk powder/TBS-T. After further washing steps, membranes were incubated for 2 min in WesternBright ECL HRP substrate (Advansta) and chemiluminescence was detected with the Amersham™ ImageQuant™ 800 biomolecular imager (Cytiva). Image analysis was performed with the ImageQuant TL software version 8.2 (GE Healthcare).

### Antibodies

**Table:** 

Target (-Conjugate) [Clone]	RRID	Host [Isotype]	Concentration/Dilution (Application)	Manufacturer (Catalog ID)
Meprin α anti-serum	custom antibody	rabbit	1:5000 (WB)	Pineda Antibody Service
Rabbit IgG H + L (HRP) [polyclonal]	AB_2313567	goat	1:5000 (WB)	Jackson ImmunoResearch (111–035-003)
Mouse IgG H + L (HRP) [polyclonal]	AB_2340295	sheep	1:5000 (WB)	Jackson ImmunoResearch (515–035-003)
Gapdh [14C10]	AB_561053	rabbit [IgG]	1:5000 (WB)	Cell Signaling Technology (2118)
CD11b (APC/Cy7) [M1/70]	AB_830641	rat [IgG2b, κ]	4 µg/mL (FC)	Revvity, BioLegend, San Diego (101225)
CD11c (FITC) [N418]	AB_313774	Armenian Hamster [IgG]	5 µg/mL (FC)	Revvity, BioLegend, San Diego (117305)
CD19 (PE/Cy7) [6D5]	AB_313654	rat [IgG2a, κ]	4 µg/mL (FC)	Revvity, BioLegend, San Diego (115519)
CD45 (BV510) [30-F11]	AB_2561392	rat [IgG2b, κ]	10 µg/mL (FC)	Revvity, BioLegend, San Diego (103137)
CD62L (FITC) [MEL-14]	AB_313092	rat [IgG2a, κ]	10 µg/mL (FC)	Revvity, BioLegend, San Diego (104405)
CD64 (APC) [X54-5/7.1]	AB_11219205	mouse [IgG1, κ]	8 µg/mL (FC)	Revvity, BioLegend, San Diego (139305)
CD3 (PerCP/Cy5.5) [17A2]	AB_1595597	rat [IgG2b, κ]	4 µg/mL (FC)	Revvity, BioLegend, San Diego (100217)
LFA-1 (APC) [H155-78]	AB_2564305	rat [IgG1, κ]	8 µg/mL (FC)	Revvity, BioLegend, San Diego (141009)
Ly6C (BV421) [HK1.4]	AB_2562177	rat [IgG2c, κ]	1 µg/mL (FC)	Revvity, BioLegend, San Diego (128031)
Ly6G (PE) [1A8]	AB_1186104	rat [IgG2a, κ]	4 µg/mL (FC)	Revvity, BioLegend, San Diego (127607)
NK1.1 (APC) [PK136]	AB_313396	mouse [IgG2a, κ]	4 µg/mL (FC)	Revvity, BioLegend, San Diego (108709)
Armenian Hamster IgG (FITC) [HTK888]	AB_2923258	Armenian Hamster [IgG]	5 µg/mL (FC)	Revvity, BioLegend, San Diego (400905)
Mouse IgG1 (APC) [MOPC-21]	AB_2888687	mouse [IgG1, κ]	8 µg/mL (FC)	Revvity, BioLegend, San Diego (400119)
Mouse IgG2a (APC) [MOPC-173]	AB_326468	mouse [IgG2a, κ]	4 µg/mL (FC)	Revvity, BioLegend, San Diego (400219)
Rat IgG1 (APC) [RTK2071]	AB_326517	rat [IgG1, κ]	8 µg/mL (FC)	Revvity, BioLegend, San Diego (400411)
Rat IgG2a (PE/Cy7) [RTK2758]	AB_326542	rat [IgG2a, κ]	4 µg/mL (FC)	Revvity, BioLegend, San Diego (400522)
Rat IgG2a (PE) [RTK2758]	AB_326530	rat [IgG2a, κ]	4 µg/mL (FC)	Revvity, BioLegend, San Diego (400507)
Rat IgG2a (FITC) [RTK2758]	AB_2736919	rat [IgG2a, κ]	10 µg/mL (FC)	Revvity, BioLegend, San Diego (400505)
Rat IgG2b (APC/Cy7) [RTK4530]	AB_326565	rat [IgG2b, κ]	4 µg/mL (FC)	Revvity, BioLegend, San Diego (400623)
Rat IgG2b (BV510) [RTK4530]	AB_3097656	rat [IgG2b, κ]	10 µg/mL (FC)	Revvity, BioLegend, San Diego (400645)
Rat IgG2b (PerCP/Cy5.5) [RTK4530]	AB_893693	rat [IgG2b, κ]	4 µg/mL (FC)	Revvity, BioLegend, San Diego (400631)
Rat IgG2c (BV421) [RTK4174]	AB_3097000	rat [IgG2c, κ]	1 µg/mL (FC)	Revvity, BioLegend, San Diego (400725)

Research Resource Identifiers (RRID); Western Blotting (WB); Flow Cytometry (FC).

### Bead-based cytokine and chemokine multiplex

Cytokine and chemokine concentrations in serum were measured with a customized LEGENDplex™ kit (BioLegend/Revvity) according to the manufacturer’s instructions as previously described (Köhling et al. 2026). Samples were measured with a FACS Symphony A1 flow cytometer (Becton Dickinson). Data analysis was conducted with the cloud-based LEGENDplex™ Data Analysis Software Suite provided by BioLegend/Revvity.

### Flow cytometry

Morphological characteristics as well as the expression of lineage and activation status-related proteins on the cell surface of leukocytes were analyzed by flow cytometry. For the analysis of whole-blood samples, 100 µL EDTA blood were diluted with 900 µL VersaLyse Lysing Solution (Beckman Coulter) for 10 min at room temperature to eliminate erythrocytes. Afterwards, cells were centrifuged at 200 g and 4 °C for 10 min and resuspended in 50 µL MACS buffer. For the analysis of isolated neutrophils, 200,000 to 500,000 cells per sample were transferred into a 96-well V-bottom plate. Cells were centrifuged at 250 g and 4 °C for 10 min and resuspended in 50 μL MACS buffer. Then, 5 μl TruStain FcX™ FcR blocking reagent (BioLegend, Revvity) were added and cells were incubated for 10 min at 4 °C in the dark. Afterwards, cells were incubated with respective antibodies diluted in MACS buffer for 15 min at 4 °C in the dark. Afterwards, cells were washed twice in ice-cold MACS buffer and fixed in 1% paraformaldehyde in MACS buffer. Measurement was performed with a FACSymphony A1 flow cytometer (Becton Dickinson). Compensation and evaluation of the data was performed using FlowJo software (Becton Dickinson).

### RNA isolation, cDNA synthesis and real-time polymerase chain reaction

Leukocytes from 0.5 mL human EDTA-whole blood were isolated by erythrocyte lysis using VersaLyse Lysing Solution (Beckman Coulter) according to the manufacturer’s instructions. Leukocytes were washed in ice-cold sterile PBS followed by RNA isolation with the NucleoSpin RNA Mini-Kit (Macherey–Nagel) and cDNA synthesis with the RevertAid First Strand cDNA Synthesis Kit (Thermo Fisher Scientific), both according to the manufacturer’s instructions. Real-time polymerase chain reaction analyses were carried out as previously reported (Köhling et al. 2026) with a qTower3 384G Real-time Thermocycler (Analytik Jena). PCR reactions were set up with Luna® Universal qPCR Master Mix (New England Biolabs) according to the manufacturer’s instructions. Melting curve analysis was run by increasing the temperature from 60 °C to 95 °C in increments of 0.5 °C. Data analysis was performed with qPCRsoft384 software version 1.2 (Analytik Jena). Relative mRNA levels were calculated by 2^−ΔΔCt^-method with Gapdh mRNA levels as reference.

### Primer

**Table:** 

Gene	Transcript ID	Sequence (5’−3’)	Amplicon (bp)	T_m_ (°C)
GAPDH	NM_002046.7	Fw: TCG GAG TCA ACG GAT TTG GT Rev: TTC CCG TTC TCA GCC TTG AC	181	82.8
MEP1A	NM_005588.3	Fw: AGC AGC TGT ACC GAT TAA GTA TCT Rev: GGT CCA AGC CTG CAG CTA AA	104	78.3

### Statistics

Statistical analysis was performed using GraphPad Prism 10.4.2 as previously described (Köhling et al. 2026). Data sets were tested for Gaussian distribution and equal variance by Shapiro–Wilk and Equal Variance test, respectively. For comparison of two-groups comprising parametrically distributed data, two-tailed unpaired t-test was conducted. In case Shapiro–Wilk test was passed but datasets failed Equal Variance test, Welch’s correction was performed. Datasets that failed normality test were analyzed by two-tailed unpaired Mann–Whitney Rank Sum test. In all cases, confidences level was set to 95% assuming statistically significant differences for *P*-values < 0.05. Parametric data sets comprising multiple groups were compared by one-way analysis of variance (one-way ANOVA) for statistical significance. Correction for multiple comparisons was conducted by Tukey’s test. Non-parametrical datasets comprising multiple groups were analyzed with Kruskal–Wallis one-way ANOVA on ranks. Correction for multiple comparisons was conducted by Dunn’s test. Curve fitting was performed by simple linear regression or non-linear regression tool. Outliers were not eliminated for regression analysis and least squares regression was chosen as fitting method. For non-linear regression, convergence criteria were set to “medium” with automatic switch to “strict” when recommended. Maximum number of iterations was set to 1000. No weighting was performed. Regression start and end were set to “automatic” and 95% confidence bands were plotted for both linear and non-linear regressions.

For the assessment of correlations between data sets that contained two discontinuous dependent parametrically distributed parameters Pearson correlation was computed. For the assessment of correlations between data sets that contained two discontinuous dependent non-parametrically distributed parameters Spearman correlation was computed.

Differences in survival were assessed by Kaplan–Meier analysis. For curve comparison Mantel-Cox test and Gehan-Breslow-Wilcoxon test were performed. Receiver Operating Characteristic analysis was performed by Wilson/Brown method with a confidence interval of 95%.

Significance was defined as *P* < 0.05 and annotated by asterisks (*). Error bars in graphs represent the standard error of the mean (SEM). Data is summarized as mean ± SEM. Differences between groups that did not reach significance level were not marked. Data were graphically visualized with GraphPad Prism v10.6.0.

## Supplementary Information

Supplementary Material 1. Fig. S1 Hematological and phenotypic analyses of K5Mα mice. (A) Red blood cell distribution width – coefficient of variation (RDW-CV), mean corpuscular hemoglobin concentration (MCHC) and hemoglobin concentrations (HGB) in control (*n*=28) and K5Mα (*n*=31) mice. (B) Lymphocyte, monocyte, eosinophil and basophil counts in control (*n*=28) and K5Mα (*n*=31) mice. (A+B) Median values are marked by horizontal red drawn-through lines. Reference ranges are marked by dotted lines and reference mean values by dashed lines. (C) Leukocyte proportions in the blood of K5Mα mice (*n*=23) and control mice (*n*=13). Mean ±SEM. Proportion of inflammatory monocytes within the monocyte population in the blood of K5Mα mice (*n*=16) and control mice (*n*=27). Median values are marked by horizontal red drawn-through lines. (D) Representative contour plots for the bone marrow analysis. (E) Correlation of G-CSF serum concentrations with the proportion of neutrophils in the blood of K5Mα mice (*n*=44). Red dashed lines mark the mean concentration and proportion detected in respective control mice. Non-linear regression (---) and respective 95% confidence band of the best-fit line (∙∙∙). (F) Superoxide (SO) and reactive oxygen species (ROS) levels in naïve purified neutrophils stimulated with serum from control (*n*=4) and K5Mα (*n*=5) mice after 30 min (SO) and 60 min (ROS). Mean ±SEM. (G) Phenotypic alterations in K5Mα mice (*n*=18) from day 0 to 18. Scores correlate with defined phenotype alterations as described in the Materials and Methods section. Days marked by black boxes indicate that mice had been sacrificed or died. Statistically significant differences (P<0.05) *are* labeled by an asterisk (*).

Supplementary Material 2. Fig. S2 G-CSF neutralization in K5Mα mice. (A) Time course of tamoxifen treatment and G-CSF-neutralizing antibody treatment. On day seven 25 µg of either an isotype control antibody (rat IgG_1_;Ctrl; *n*=9) or a G-CSF neutralizing antibody (α G-CSF; *n*=9) diluted in sterile saline solution were injected intraperitoneal. On days eight, nine and ten - 24, 48 and 72 h after antibody treatment – blood and bone marrow of three mice per group were analyzed. (B) G-CSF levels in the serum, (C) white blood cell counts and (D) neutrophil counts in the blood as well as proportions of premature (CD11b+ Ly6G-; CD11b+ Ly6G^low^) and mature (CD11b+ Ly6G^high^) neutrophils in the bone marrow of K5Mα mice treated with control (Ctrl) or G-CSF neutralizing (α G-CSF) antibody. Statistically significant differences (P<0.05) were tested by two-tailed unpaired t-test and labeled by an asterisk (*). Graphical illustration in (A) was created with BioRender.com

Supplementary Material 3. Fig. S3 Characterization of LMα mice. (A) Scheme illustrates the genotype of transgenic mice designated as “control” and “LMα”. Both strains were bred on a C57BL7/6J background harboring a homozygous (+/+) insertion in the Rosa26 locus composed of a CAG-promotor (CAG), a loxP site (►)-flanked neomycin resistance (Neo)/STOP cassette, a cDNA sequence encoding HA-tagged murine meprin α (Mep1a-HA), an internal ribosomal entry site (IRES) and a cDNA sequence encoding enhanced green fluorescent protein (EGFP). LMα mice, but not control mice, also carry heterozygous (+/-) downstream of the Lyz2 promoter a substitution of the Lyz2 gene by a gene encoding cre recombinase. (B) Western blot detection of meprin α in the bone marrow of K5Mα (CreER^T2^+) and LMα (CreER^T2^+) as well as respective control (CreER^T2^-) mice. Gapdh was detected as reference. (C) Age of LMα (*n*=45) and respective control (*n*=44) mice in weeks. Violin plots display data distribution and mark median values (horizontal drawn-through line). (D) Hematocrit (HCT), (E) red blood cell count (RBC), (F) mean corpuscular volume (MCV), (G) red blood cell distribution width– coefficient of variation (RDW-CV), (H) hemoglobin concentration (HGB), (I) mean corpuscular hemoglobin (MCH), (J) mean corpuscular hemoglobin concentration (MCHC), (K) platelet count (PLT), (L) mean platelet volume (MPV), (M) white blood cell count (WBC), (N) neutrophil count, (O) lymphocyte count, (P) monocyte count, (Q) eosinophil count and (R) basophil count measured in EDTA-whole blood samples from control and LMα mice. Data points show respective values detected in individual control (*n*=44) and LMα (*n*=46) mice. Violin plots display data distribution and mark median values (horizontal drawn-through line). Reference ranges marked by black dotted lines and mean values are marked by black dashed lines. (S) Proportion of B cells (CD19+), T cells (CD3+ NK1.1-), neutrophils (CD11b+ Ly6G+), monocytes (CD11b+ Ly6G-, NK1.1-), NK cells (NK1.1+ CD3-) and NK T cells (NK1.1+ CD3+) within CD45+ leukocytes in the peripheral blood of control and LMα mice measured by flow cytometry. Data points show respective values detected in individual control (*n*=16-22) and K5Mα (*n*=20-22) mice. Bar charts and error bars display mean values and respective standard errors of the mean (SEM). (T) Proportion of CD11b+ Ly6G-, CD11b+ Ly6G^low^ and CD11b+ Ly6G^high^ CD45+ leukocytes in the bone marrow of control and LMα mice measured by flow cytometry. Data points show respective values detected in individual control (*n*=20) and K5Mα (*n*=22) mice. Bar charts and error bars display mean values and respective SEM. (U) Heatmap displays log2-transformed concentrations of interleukin-1 alpha (IL-1α), interleukin-6 (IL-6), interleukin-18 (IL-18), tumor necrosis factor alpha (TNFα), granulocyte colony stimulating factor (G-CSF), C-X-C motif chemokine ligand 1 (CXCL1) and CC motif chemokine ligand 2 (CCL2) in the serum of control (n = 28) and LMα mice (n = 30). Higher (red) and lower (blue) concentrations are highlighted by color gradients. Cytokine/chemokine levels were quantified by a custom-generated bead-based multiplex assay (LEGENDplex, Revvity). Statistically significant differences (P<0.05) were tested by two-tailed unpaired t-test and labeled by an asterisk (*). Graphical illustration in (A) was created with BioRender.com

Supplementary Material 4. Fig. S4: Meprin α urine protein levels and mRNA levels in leukocytes of non-SIRS and SIRS patients. (A) Urinary meprin α concentrations in non-SIRS (*n*=5) and SIRS (*n*=5) patients. Median values are marked by horizontal red dashed lines. In the urine of two non-SIRS patients and one SIRS patient meprin α was not detectable. (B) Relative meprin α mRNA levels in peripheral blood leukocytes of non-SIRS (*n*=5) and SIRS (*n*=5) patients normalized to respective Gapdh mRNA levels by ΔΔCt-method. In leukocytes of four non-SIRS and four SIRS patients meprin α mRNA was not detectable

Supplementary Material 5. Table S1 Clinical parameters of the study cohort at the time of admission to the intensive care unit (ICU). Annotated are median values with 10^th^ and 90^th^ percentiles (P10/P90). Statistically significant differences between patients who did not develop a systemic inflammatory response syndrome (SIRS) and those who did develop SIRS were assessed by two-tailed unpaired Mann-Whitney test. Spearman r-values were computed for correlation of serum meprin α levels and respective clinical parameters of the both groups combined. Two-tailed P-values are listed. Area under the curve (AUC) values were computed from Receiver Operating Curve analysis and respective P-values are listed.

Supplementary Material 6.

## Data Availability

All data needed to evaluate the conclusions in the paper are present in the paper and/or the Supplementary Materials. Data sets presented in this study are available on reasonable request from the corresponding author.

## Abbreviations

ICU: Intensive care unit
K5Mα: Transgenic mouse line C57BL/6J-KRT5^tm1.1-CreERT2^;ROSA26^Stop-mMep1a^
LMα: Transgenic mouse line Lyz2^tm1(Cre)lfo^;Rosa26^Stop-mMep1a^
M-CASS: Modified Mouse Clinical Assessment Score for Sepsis
ROS: Reactive oxygen species
SIRS: Systemic inflammatory response syndrome
SO: Superoxide
