# Supplementary figures and images for: Serum meprin α levels for the detection of systemic inflammatory response syndrome
Source: Mol Med. 2026 Jul 18;32:113. doi: 10.1186/s10020-026-01570-w (PMC13380840; doi:10.1186/s10020-026-01570-w)

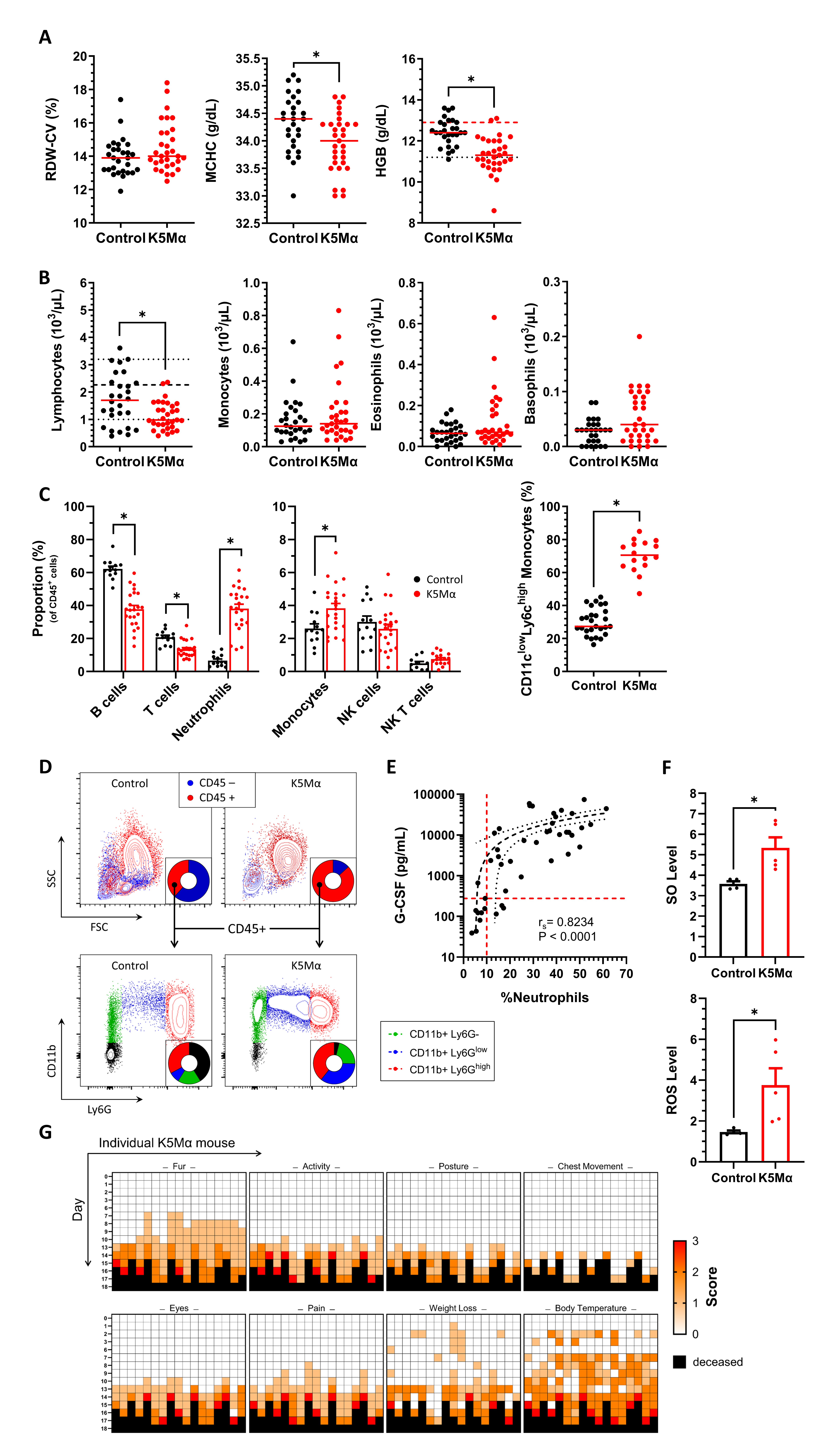

Supplement: Supplementary file 1 — Supplementary Material 1. Fig. S1 Hematological and phenotypic analyses of K5Mα mice. (A) Red blood cell distribution width – coefficient of variation (RDW-CV), mean corpuscular hemoglobin concentration (MCHC) and hemoglobin concentrations (HGB) in control (n=28) and K5Mα (n=31) mice. (B) Lymphocyte, monocyte, eosinophil and basophil counts in control (n=28) and K5Mα (n=31) mice. (A+B) Median values are marked by horizontal red drawn-through lines. Reference ranges are marked by dotted lines and reference mean values by dashed lines. (C) Leukocyte proportions in the blood of K5Mα mice (n=23) and control mice (n=13). Mean ±SEM. Proportion of inflammatory monocytes within the monocyte population in the blood of K5Mα mice (n=16) and control mice (n=27). Median values are marked by horizontal red drawn-through lines. (D) Representative contour plots for the bone marrow analysis. (E) Correlation of G-CSF serum concentrations with the proportion of neutrophils in the blood of K5Mα mice (n=44). Red dashed lines mark the mean concentration and proportion detected in respective control mice. Non-linear regression (---) and respective 95% confidence band of the best-fit line (∙∙∙). (F) Superoxide (SO) and reactive oxygen species (ROS) levels in naïve purified neutrophils stimulated with serum from control (n=4) and K5Mα (n=5) mice after 30 min (SO) and 60 min (ROS). Mean ±SEM. (G) Phenotypic alterations in K5Mα mice (n=18) from day 0 to 18. Scores correlate with defined phenotype alterations as described in the Materials and Methods section. Days marked by black boxes indicate that mice had been sacrificed or died. Statistically significant differences (P<0.05) are labeled by an asterisk (*). [file 10020_2026_1570_MOESM1_ESM.tif]

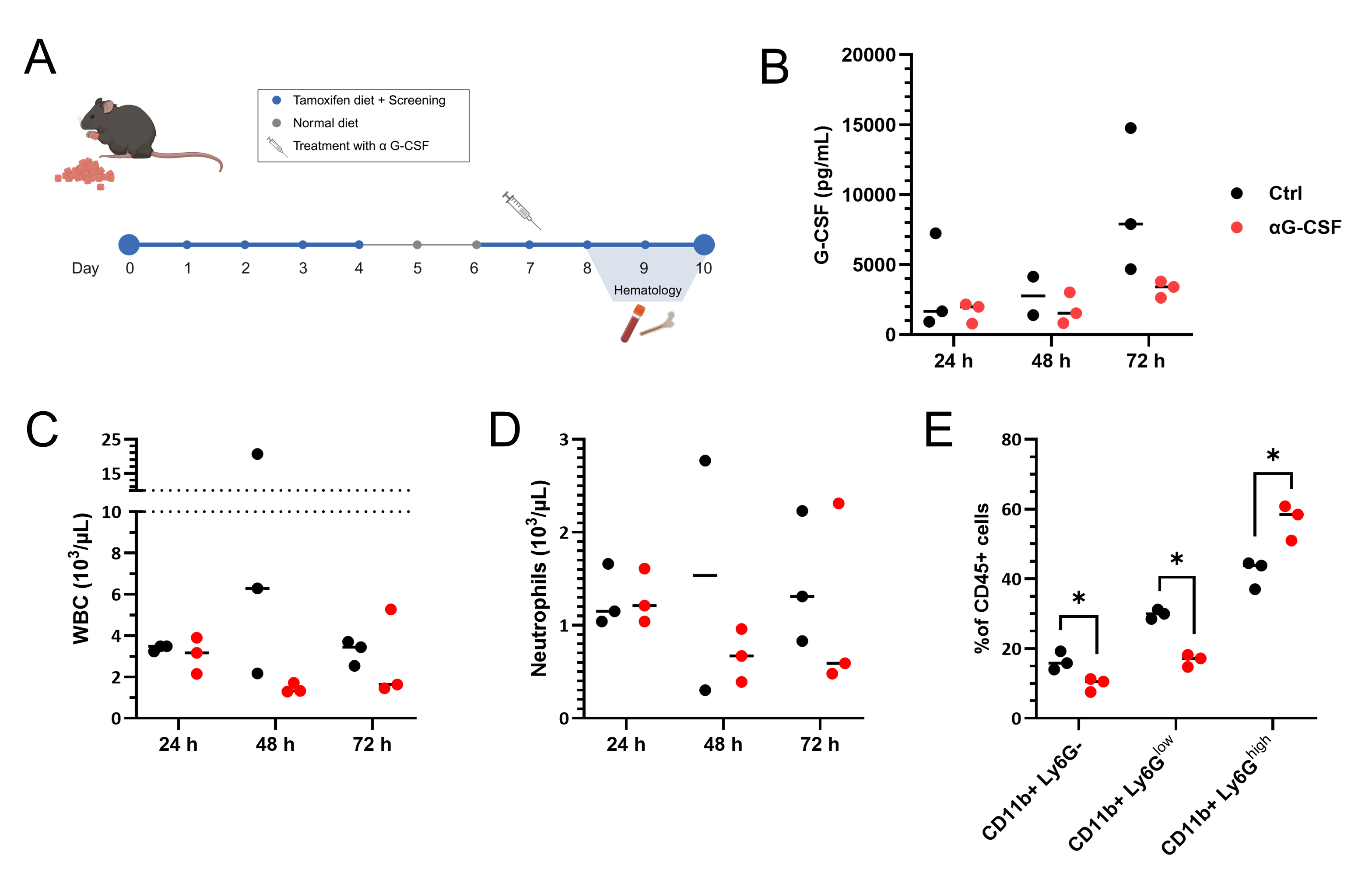

Supplement: Supplementary file 2 — Supplementary Material 2. Fig. S2 G-CSF neutralization in K5Mα mice. (A) Time course of tamoxifen treatment and G-CSF-neutralizing antibody treatment. On day seven 25 µg of either an isotype control antibody (rat IgG1;Ctrl; n=9) or a G-CSF neutralizing antibody (α G-CSF; n=9) diluted in sterile saline solution were injected intraperitoneal. On days eight, nine and ten - 24, 48 and 72 h after antibody treatment – blood and bone marrow of three mice per group were analyzed. (B) G-CSF levels in the serum, (C) white blood cell counts and (D) neutrophil counts in the blood as well as proportions of premature (CD11b+ Ly6G-; CD11b+ Ly6Glow) and mature (CD11b+ Ly6Ghigh) neutrophils in the bone marrow of K5Mα mice treated with control (Ctrl) or G-CSF neutralizing (α G-CSF) antibody. Statistically significant differences (P<0.05) were tested by two-tailed unpaired t-test and labeled by an asterisk (*). Graphical illustration in (A) was created with BioRender.com [file 10020_2026_1570_MOESM2_ESM.tif]

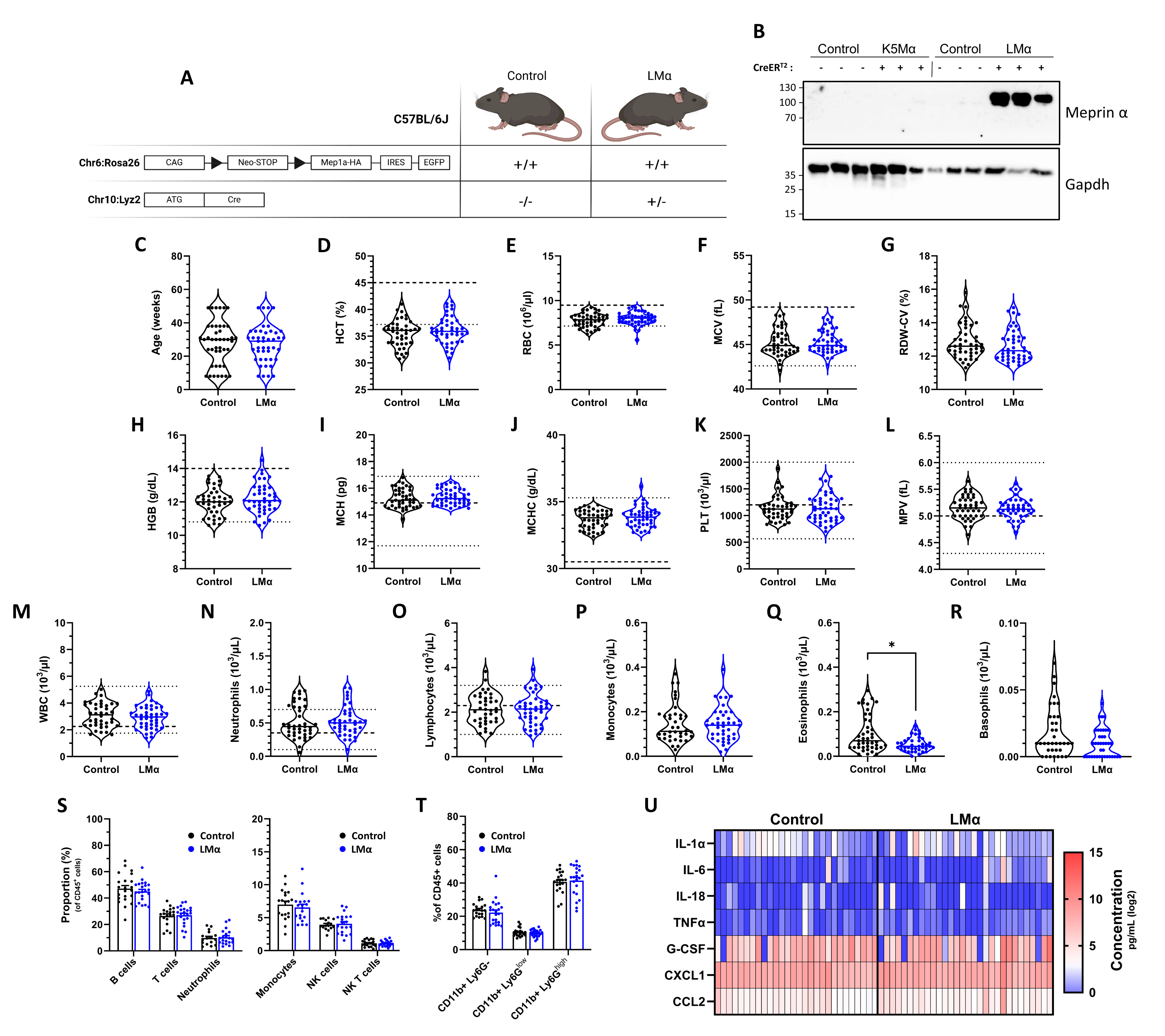

Supplement: Supplementary file 3 — Supplementary Material 3. Fig. S3 Characterization of LMα mice. (A) Scheme illustrates the genotype of transgenic mice designated as “control” and “LMα”. Both strains were bred on a C57BL7/6J background harboring a homozygous (+/+) insertion in the Rosa26 locus composed of a CAG-promotor (CAG), a loxP site (►)-flanked neomycin resistance (Neo)/STOP cassette, a cDNA sequence encoding HA-tagged murine meprin α (Mep1a-HA), an internal ribosomal entry site (IRES) and a cDNA sequence encoding enhanced green fluorescent protein (EGFP). LMα mice, but not control mice, also carry heterozygous (+/-) downstream of the Lyz2 promoter a substitution of the Lyz2 gene by a gene encoding cre recombinase. (B) Western blot detection of meprin α in the bone marrow of K5Mα (CreERT2+) and LMα (CreERT2+) as well as respective control (CreERT2-) mice. Gapdh was detected as reference. (C) Age of LMα (n=45) and respective control (n=44) mice in weeks. Violin plots display data distribution and mark median values (horizontal drawn-through line). (D) Hematocrit (HCT), (E) red blood cell count (RBC), (F) mean corpuscular volume (MCV), (G) red blood cell distribution width– coefficient of variation (RDW-CV), (H) hemoglobin concentration (HGB), (I) mean corpuscular hemoglobin (MCH), (J) mean corpuscular hemoglobin concentration (MCHC), (K) platelet count (PLT), (L) mean platelet volume (MPV), (M) white blood cell count (WBC), (N) neutrophil count, (O) lymphocyte count, (P) monocyte count, (Q) eosinophil count and (R) basophil count measured in EDTA-whole blood samples from control and LMα mice. Data points show respective values detected in individual control (n=44) and LMα (n=46) mice. Violin plots display data distribution and mark median values (horizontal drawn-through line). Reference ranges marked by black dotted lines and mean values are marked by black dashed lines. (S) Proportion of B cells (CD19+), T cells (CD3+ NK1.1-), neutrophils (CD11b+ Ly6G+), monocytes (CD11b+ Ly6G-, NK1.1-), NK [file 10020_2026_1570_MOESM3_ESM.tif]

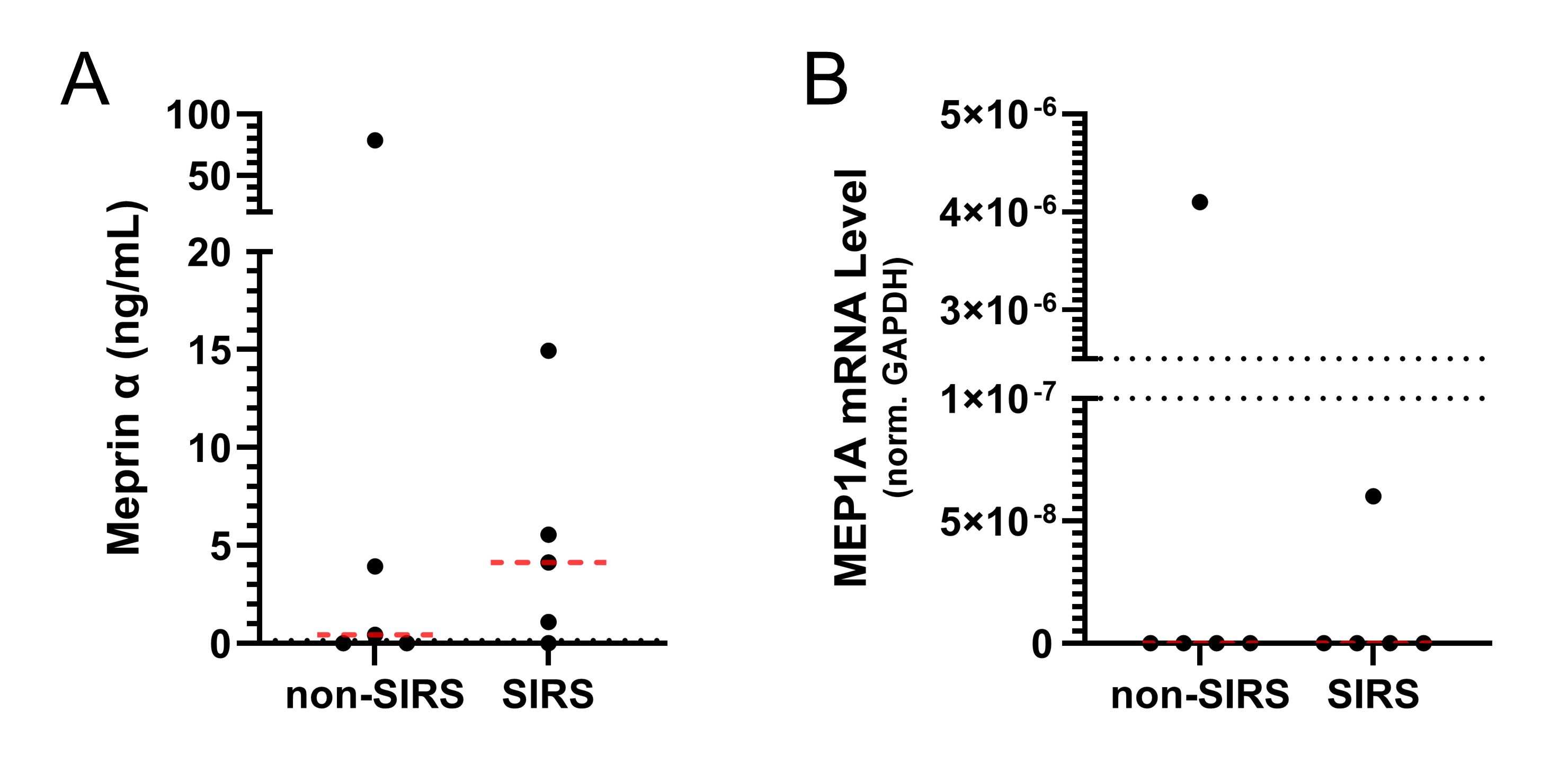

Supplement: Supplementary file 4 — Supplementary Material 4. Fig. S4: Meprin α urine protein levels and mRNA levels in leukocytes of non-SIRS and SIRS patients. (A) Urinary meprin α concentrations in non-SIRS (n=5) and SIRS (n=5) patients. Median values are marked by horizontal red dashed lines. In the urine of two non-SIRS patients and one SIRS patient meprin α was not detectable. (B) Relative meprin α mRNA levels in peripheral blood leukocytes of non-SIRS (n=5) and SIRS (n=5) patients normalized to respective Gapdh mRNA levels by ΔΔCt-method. In leukocytes of four non-SIRS and four SIRS patients meprin α mRNA was not detectable [file 10020_2026_1570_MOESM4_ESM.tif]
