# Supplementary material for: Serum meprin α levels for the detection of systemic inflammatory response syndrome
Source: Mol Med. 2026 Jul 18;32:113. doi: 10.1186/s10020-026-01570-w (PMC13380840; doi:10.1186/s10020-026-01570-w)
Supplement: Supplementary file 5 — Supplementary Material 5. Table S1 Clinical parameters of the study cohort at the time of admission to the intensive care unit (ICU). Annotated are median values with 10th and 90th percentiles (P10/P90). Statistically significant differences between patients who did not develop a systemic inflammatory response syndrome (SIRS) and those who did develop SIRS were assessed by two-tailed unpaired Mann-Whitney test. Spearman r-values were computed for correlation of serum meprin α levels and respective clinical parameters of the both groups combined. Two-tailed P-values are listed. Area under the curve (AUC) values were computed from Receiver Operating Curve analysis and respective P-values are listed. [file 10020_2026_1570_MOESM5_ESM.docx]

**Table S1 Clinical parameters of the study cohort at the time of admission to the intensive care unit (ICU).** Annotated are median values with 10^th^ and 90^th^ percentiles (P10/P90). Statistically significant differences between patients who did not develop a systemic inflammatory response syndrome (SIRS) and those who did develop SIRS were assessed by two-tailed unpaired Mann-Whitney test. Spearman r-values were computed for correlation of serum meprin α levels and respective clinical parameters of the both groups combined. Two-tailed P-values are listed. Area under the curve (AUC) values were computed from Receiver Operating Curve analysis and respective P-values are listed.

| **Parameter** | **non-SIRS**  **Median**  **(P10/P90)** | **SIRS**  **Median**  **(P10/P90)** | **Mann-Whitney**  **P-Value** | **Spearman r**  **(vs. Meprin α)** | **Spearman**  **P-Value** | **AUC**  **(P-Value)** |
| --- | --- | --- | --- | --- | --- | --- |
| Meprin α (ng/mL) | 0.03  (0.00/0.13) | 0.35  (0.02/1.23) | <0.0001 |  |  | 0.8612  (<0.0001) |
| CRP (mg/L) | 68.7  (3.6/171.2) | 157.0  (42.6/357.0) | 0.0001 | 0.3403 | 0.0237 | 0.8284  (0.0002) |
| PCT (µg/L) | 0.29  (0.06/8.48) | 0.73  (0.17/5.51) | 0.1115 | 0.5326 | 0.0004 | 0.6496  (0.1094) |
| PTX3 (pg/mL) | 21,757  (6,780/73,388) | 29,730  (11,378/74,247) | 0.1790 | 0.3428 | 0.0170 | 0.6171  (0.1738) |
| WBC (x10³/µL) | 8.9  (6.4/14.2) | 15.1  (9.7/28.0) | <0.0001 | 0.4908 | 0.0004 | 0.8383  (<0.0001) |
| IL-6 (pg/mL) | 42.7  (8.5/552.9) | 123.4  (24.9/498.9) | 0.0171 | 0.3946 | 0.0060 | 0.7042  (0.0177) |
| IL-18 (pg/mL) | 157.5  (31.6/446.5) | 335.3  (96.6/1,703.0) | 0.0258 | 0.3246 | 0.0244 | 0.6915  (0.0261) |
| G-CSF (pg/mL) | 76.5  (20.9/1,141.0) | 125.1  (26.0/2,069.0) | 0.2853 | 0.1200 | 0.4165 | 0.5935  (0.2775) |
| CXCL1 (pg/mL) | 107.2  (41.7/239.1) | 227.0  (52.6/427.9) | 0.0171 | 0.2674 | 0.0662 | 0.7042  (0.0177) |
| CXCL8 (pg/mL) | 46.0  (12.6/304.6) | 143.2  (29.8/590.1) | 0.0029 | 0.5629 | <0.0001 | 0.7514  (0.0035) |
| CCL2 (pg/mL) | 272.3  (107.9/1,052.0) | 464.2  (126/1,474) | 0.0421 | 0.2074 | 0.1572 | 0.6751  (0.0419) |
| HCT (%) | 30.2  (24.6/37.9) | 27.0  (24.0/38.7) | 0.2217 | -0.2421 | 0.0973 | 0.6062  (0.2174) |
| HGB (g/L) | 10.3  (7.6/12.9) | 8.7  (7.7/13.2) | 0.0717 | -0.3191 | 0.0270 | 0.6552  (0.0714) |
| PLT (x10^9^/µL) | 199.5  (89.9/408.0) | 203.0  (75.0/360.0) | 0.5367 | -0.2515 | 0.0881 | 0.5545  (0.5295) |
| Quick (%) | 80  (50/97) | 75  (52/105) | 0.3565 | -0.3037 | 0.0401 | 0.5823  (0.3502) |
| PTT (s) | 28.5  (24.8/51.5) | 30.0  (25.9/39.2) | 0.3851 | 0.3815 | 0.0088 | 0.5774  (0.3800) |
| Glucose (mg/dL) | 122  (83/218.0) | 149  (77/209) | 0.2598 | 0.09553 | 0.5183 | 0.5980  (0.2549) |
| Lactate (mmol) | 1.0  (0.5/1.9) | 1.1  (0.7/3.6) | 0.2126 | 0.2245 | 0.1250 | 0.6080  (0.2096) |
| GFR CDK-EPI (mL/min/1.73m²) | 74.0  (26.8/115.6) | 58.0  (17.0/103.0) | 0.1402 | -0.3350 | 0.0228 | 0.6082  (0.2156) |
| Creatinine | 79.8  (43.7/215.4) | 103.0  (63.4/285.0) | 0.1191 | 0.3411 | 0.0203 | 0.6374  (0.1157) |
| Albumin (g/L) | 31.4  (25.5/36.5) | 28.7  (24.3/35.0) | 0.0560 | -0.01371 | 0.9367 | 0.6938  (0.0484) |
| ALT (IU/L) | 14.7  (6.0/1,281.0) | 27.2  (6.0/506.0) | 0.5872 | 0.2355 | 0.3764 | 0.5873  (0.5604) |
| AST (IU/L) | 32.4  (18.6/211.0) | 64.1  (20.6/327.4) | 0.2164 | 0.6184 | <0.0001 | 0.6223  (0.2106) |
| Bilirubin | 8.2  (2.9/14.3) | 9.4  (4.1/118.2) | 0.2315 | 0.3888 | 0.0209 | 0.6209  (0.2220) |
| µ-GT (IU/L) | 36.0  (9.2/133.0) | 98.0  (21.0/645.0) | 0.0272 | 0.3001 | 0.0534 | 0.6991  (0.0279) |
