# Supplementary material for: Serum meprin α levels for the detection of systemic inflammatory response syndrome
Source: Mol Med. 2026 Jul 18;32:113. doi: 10.1186/s10020-026-01570-w (PMC13380840; doi:10.1186/s10020-026-01570-w)
Supplement: Supplementary file 6 — Supplementary Material 6. [file 10020_2026_1570_MOESM6_ESM.pptx]

## Slide 1
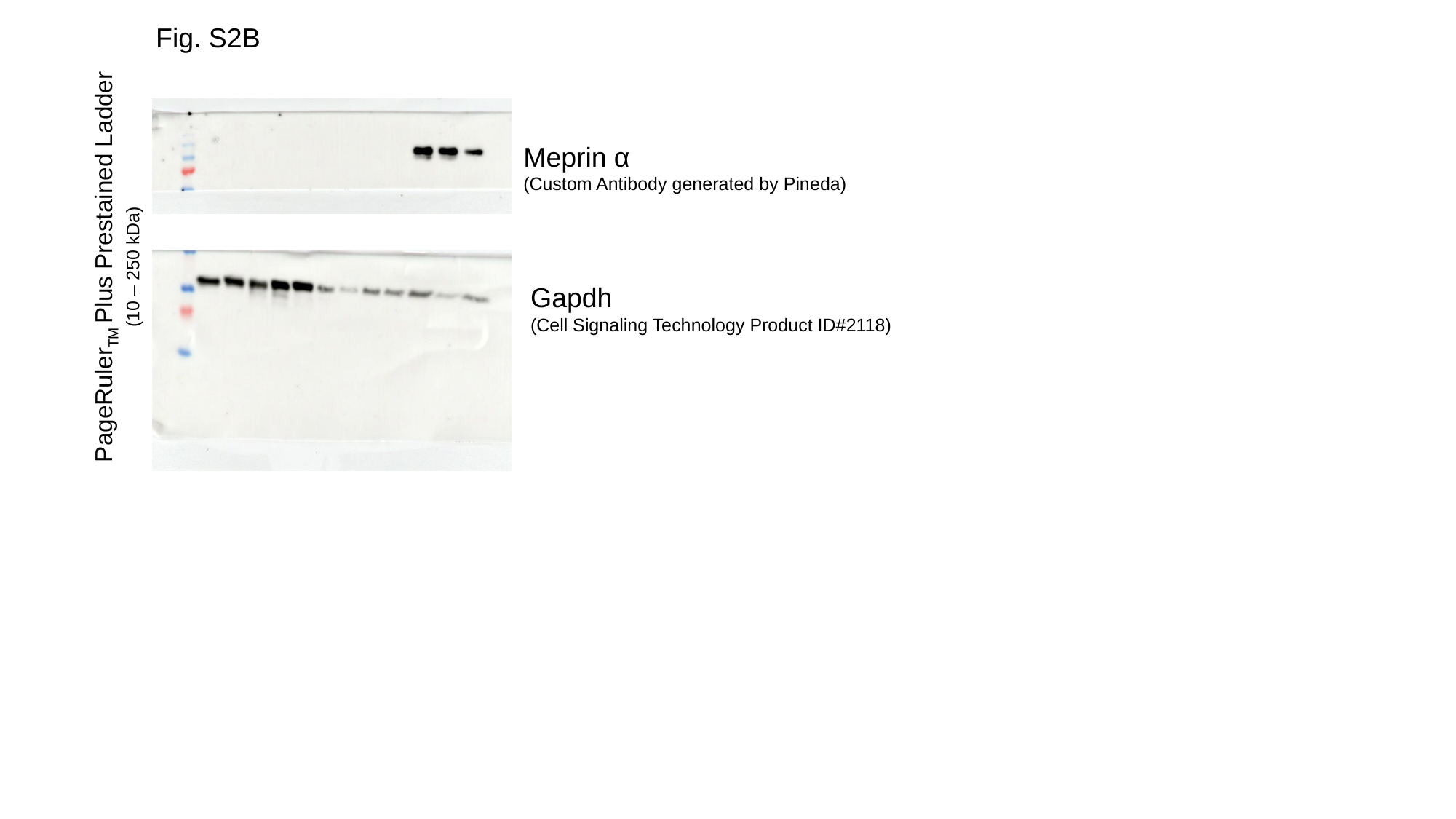

Fig. S2B
Meprin α
(Custom Antibody generated by Pineda)
PageRulerTM Plus Prestained Ladder
(10 – 250 kDa)
Gapdh
(Cell Signaling Technology Product ID#2118)
